# Electrical transport properties of an isolated CdS microrope composed of twisted nanowires

**DOI:** 10.1186/s11671-015-0734-5

**Published:** 2015-01-28

**Authors:** Gui-Feng Yu, Miao Yu, Wei Pan, Wen-Peng Han, Xu Yan, Jun-Cheng Zhang, Hong-Di Zhang, Yun-Ze Long

**Affiliations:** Collaborative Innovation Center for Low-Dimensional Nanomaterials and Optoelectronic Devices, Qingdao University, Qingdao, 266071 People’s Republic of China; College of Physics, Qingdao University, Qingdao, 266071 People’s Republic of China; College of Science and Information, Qingdao Agricultural University, Qingdao, 266109 People’s Republic of China; Department of Mechanical Engineering, Columbia University, New York, NY 10027 USA; College of Chemistry and Pharmaceutical Sciences, Qingdao Agricultural University, Qingdao, 266109 People’s Republic of China; Collaborative Innovation Center for Marine Biomass Fibers, Materials and Textiles of Shandong Province, State Key Laboratory Cultivation Base of New Fiber Materials and Modern Textile, Qingdao University, Qingdao, 266071 People’s Republic of China

**Keywords:** CdS microrope, Space-charge-limited current, Coulomb blockade

## Abstract

CdS is one of the important II-VI group semiconductors. In this paper, the electrical transport behavior of an individual CdS microrope composed of twisted nanowires is studied. It is found that the current–voltage (*I*-*V*) characteristics show two distinct power law regions from 360 down to 60 K. Space-charge-limited current (SCLC) theory is used to explain these temperature- and electric-field-dependent *I-V* curves. The *I-V* data can be well fitted by this theory above 100 K, and the corresponding carrier mobility, trap energy, and trap concentration are also obtained. However, the *I-V* data exhibit some features of the Coulomb blockade effect below 80 K.

## Background

One-dimensional (1D) semiconductor nanostructures (nanotubes, nanorods, nanowires, nanobelts, etc.) have gained tremendous attention within the last two decades due to their unique electronic, optical, and mechanical properties. Among the huge variety of 1D nanostructures, CdE (E = S, Se, and Te) 1D nanostructures have attracted much attention for their potential applications in solar cells [1], biosensors [2], electrochemical detection [3], and photocathodes [4]. In order to fulfill these potential applications, it is very essential to properly identify some physical characteristics which play important roles on electrical transport characteristics such as conductivity, *I*-*V* characteristic, and carrier mobility. Moreover, the extractions of material parameters (i.e., carrier mobility and trap energy) rely on analysis with specific models.

In the past decades, various theoretical models such as Fowler-Nordheim field-emission tunneling [5], Luttinger liquid theory [6], Wigner crystal model [7], variable range hopping (VRH) theory [8], fluctuation-induced tunneling (FIT) theory [9], scaling theory [10], space-charge-limited current (SCLC) theory [11], phonon-assisted tunneling theory [12], and Coulomb blockade effect [13] have been used to explain the conduction mechanism of such quasi-one-dimensional (quasi-1D) inhomogeneous structures. However, it is still an open issue how to explain the nonlinear *I*-*V* characteristics for the complexities of the structure and conduction mechanism in 1D nanofibers [14]. For instance, the non-ohmic *I*-*V* characteristic curve of single-wall carbon nanotube network was measured at 7 K, and the non-ohmic regime could be fitted well by the FIT model, indicating the importance of inter-tubular contacts or inherent energy barriers inside the tubes [15]. However, Kaiser et al. fitted the same curve to the calculated behavior with fluctuation-assisted tunneling and thermal activation model, giving a good account of the feature of the *I*-*V* curve [16]. In addition, the electrical transport mechanism of nano-CdS has been discussed by various theories [17-19]. For example, the single CdS nanowire synthesized by aqueous chemical growth showed a high electrical conductivity of 0.82 S cm^−1^ at room temperature and a small band gap of 0.055 eV, and the resistance of the nanowire increased exponentially with decreasing temperature, namely, the temperature dependence of resistance followed the typical thermal activation model [17]. The rectifying characteristics of Cu/CdS/SnO_2_/In-Ga structure were investigated in the temperature range of 130 to 325 K, indicating that the mechanism of charge transport was performed by tunneling among interface states/traps or dislocations intersecting the space charge region [18]. Analysis of the voltage and temperature dependencies of the SCLC theory in n-type CdS nanowire showed that the nanowire surface traps were exponentially distributed in energy with a characteristic depth about 0.28 ± 0.04 eV, showing that the surface traps were an essential ingredient for proper understanding of SCLC in nanowires [19].

There are also many reasons that the SCLC theory could be used in CdS microrope; the following are two of them. One is that for semiconductor nanowires that are intrinsic or depleted of charge carriers, one would expect to observe SCLC when the nanowire resistance greatly exceeds the contact resistance [19]. In the present article, the room-temperature conductivity of the measured nanowire is about 2.9 × 10^−4^ S cm^−1^, and the resistance of the CdS microrope is about 320 MΩ at room temperature, while the resistance of the Pt microlead is 2 kΩ using the widely recognized conductivity of 2 × 10^3^ S cm^−1^ for the focused ion beam (FIB) deposited Pt film. Accordingly, for the two-probe method, the contact resistance and the microleads' resistance can be ignored by contrasting to the nanowire's resistance [20,21]. The other is that the total number of surface traps can dominate over the total number of traps when the dimension reduces to the nanoscale. Moreover, the adsorbates on the nanowire surface can capture the free carriers and modify the electrostatic profile inside the nanowire; accordingly, the charges trapped at the nanowire surface can greatly influence the SCLC [22].

In this paper, the *I-V* behavior of an isolated CdS microrope composed of twisted nanowires has been measured from 360 down to 60 K. The electronic conduction mechanism is attempted to be discussed based on the SCLC theory. It is proposed that the conduction mechanism could be attributed to the SCLC theory from 360 to 100 K. Nevertheless, the current is near zero below 80 K and around zero bias, which could be attributed to Coulomb blockade transport [23], because electron–electron interaction should also be taken into account especially at low temperatures in quasi-1D systems where electron states are more localized due to confinement effect or disorder [24].

## Methods

The CdS microropes composed of twisted nanowires were prepared by a simple aqueous chemical growth route [25]. At first, 0.032 g Cd(CH_3_COO)_2_ · 2H_2_O was dissolved into 120 ml 35 mol% aqueous solution of ethylenediamine at room temperature. Then, a stoichiometric amount of Na_2_S · 9H_2_O was added to the solution under vigorous stirring. After kept out of light and heated to 50°C with moderate stirring until the milk-white mixture gradually turned to a little yellow about 2 days later, it was continuously heated at 60°C with stirring for a long term up to about 6 days. The final product was obtained by centrifugation and washed with distilled water and ethanol for several times. At last, the twisted CdS microropes composed of nanowires with diameters of 6 to 10 nm were prepared.

The obtained samples were characterized by scanning electron microscopy (SEM; JEOL JSM-6700 F, JEOL Ltd., Akishima, Tokyo, Japan) and transmission electron microscopy (TEM; JEOL JEM-2100 F). A pair of platinum micro-leads on isolated CdS microrope was fabricated by FIB (FEI Company, Hillsboro, OR, USA) deposition. The *I*-*V* characteristics of a section of the microrope between two micro-leads were measured by a Physical Property Measurement System (PPMS, Quantum Design, San Diego, CA, USA) by applying bias voltage from −6.0 to 6.0 V with a step of 0.05 V.

## Results and discussion

### *I*-*V* characteristic curves

**Figure 1:**
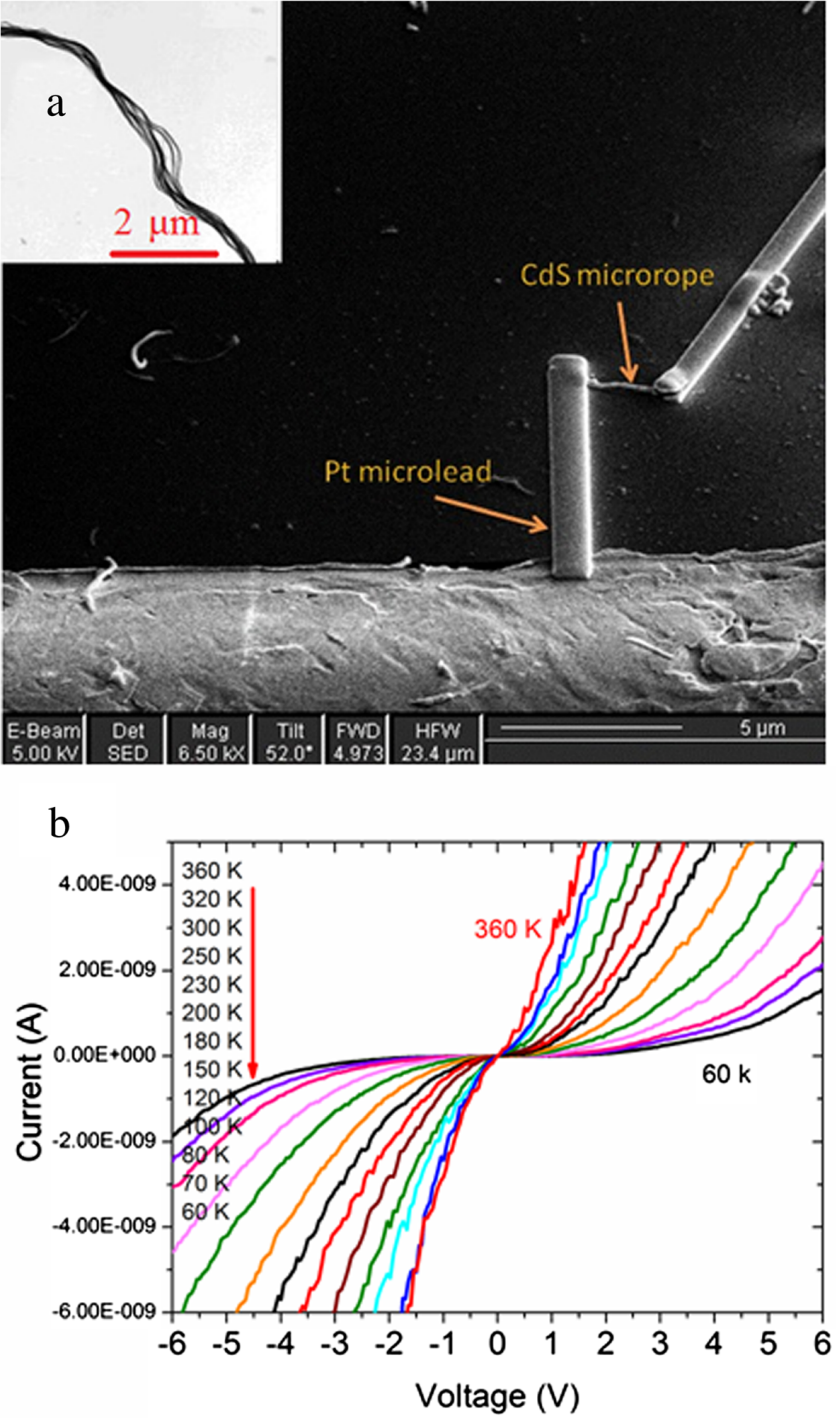
**SEM and TEM images and** ***I-V*** **characteristic curves of the isolated CdS microrope. (a)** SEM image of an isolated CdS microrope and a pair of Pt microleads fabricated with focused ion beam deposition. The inset shows the TEM image of the CdS microrope, which is composed of twisted CdS nanowires. **(b)** *I-V* characteristic curves of the isolated CdS microrope at different temperatures from 360 down to 60 K; the curves are symmetric.

The as-grown flexible CdS nanowires with diameter of 6 to 10 nm can be spontaneously self-assembled into inter-strand microropes with a spirally twisted structural conformation, and the diameter of the CdS microrope depends on the number of the wires assembled into the rope, which can be controlled just by adjusting the ligand concentration [25]. Figure 1a shows the SEM picture of an isolated CdS microrope with a width of 250 to 300 nm. The length of the CdS microrope between the two Pt microleads is about 1.7 μm. The inset of Figure 1a shows the TEM image of CdS microrope, indicating that it is composed of twisted nanowires. Figure 1b shows the typical *I*-*V* characteristics of the twisted CdS microrope covering a wide temperature range from 360 down to 60 K. It is evident that the curves are symmetric and can be divided into two regions for a given temperature. The curves show Ohm's characteristics in the lower voltage region and the SCLC characteristics at higher voltage. In addition, it is interesting to see that there is little current that can flow through the microrope at a lower bias in the temperature range from 80 to 60 K.

### SCLC theory

The SCLC theory was first discussed by Mott and Gurney [26] in 1940 for a trap-free insulator. It is based on the barrier at the metal electrode-nanowire interfaces, using in the condition that the number of injected charge is higher than the number of neutralized thermal free carriers recently [26-28]. Now, it has been used in many systems. For example, a transition from linear *I*-*V* behavior at a low bias to a SCLC behavior at a large bias has been found by Xu et al. in unintentionally doped GaSb nanowires, showing that the trap energy distribution in the nanowires has been reduced after thermal annealing [29]. Kirchartz et al. discussed the influence of charged defects on the information derived from fitting space-charge-limited current models to the experimental data [30]. Simpkins et al. exploited this theory to extract size-dependent carrier densities and demonstrated surface-dominated behavior for individual heterostructure AlGaN/GaN nanowires [31]. Cheon et al. studied diketopyrrolopyrrole-based polymers (PDPPDTSE) using the SCLC theory and time-off-light (TOL) methods; the mobility of the hole-only device based on PDPPDTSE was found to be dependent upon the electric field over the range of 10^−3^ to 10^−2^ cm^2^ V^−1^ s^−1^ [32].

**Figure 2:**
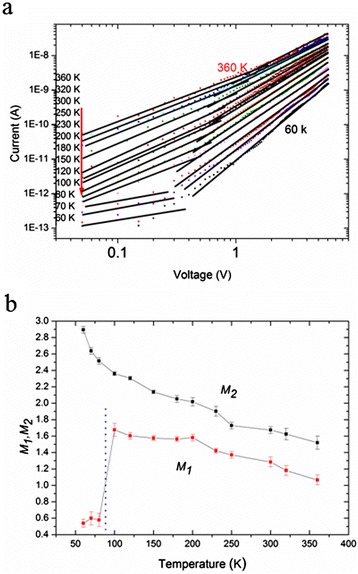
**Log-log plots of the** ***I-V*** **curves and the fitting parameters. (a)** Log-log plots of the *I-V* curves of the individual CdS microrope at different temperatures. The symbols are the experimental points, while the solid lines are the fitting lines to *I* ∞ *V* ^*M*^ of the SCLC model. **(b)** The fitting parameters *M*_2_ (at higher voltages) of the SCLC model (*I* ∞ *V* ^*M*^) and *M*_1_ (at lower voltages) of Ohm's characteristic from 360 down to 60 K.

SCLC is always used in metal–semiconductor-metal sandwich structure to discuss the conduction mechanism now. The *I*-*V* characteristic of the SCLC theory can be expressed as *I ∞ V*^*M*^, where the exponent *M* (≥1) is directly related to the depth of the trap state distribution under the conduction band. The presence of traps can not only reduce the magnitude of space-charge-limited currents but also distort the shape of the *I*-*V* curve from an ideal square law to a much higher power dependence on voltage [28]. Thus, the particular shape of the *I*-*V* characteristic curve can be used to determine the energy distribution of traps. For instance, *M* = 2 indicates that the material is in an ideal trap-free state, and *M* > 2 indicates that the material is in a trap state [27]. Figure 2a shows the log-log plots of Figure 1b. The symbols are experimental points, while the solid lines are fitting lines to the expression *I* ∞ *V*^*M*^. According to Figure 2a, it is evident that there exists a voltage *V*_i_, which represents the transition from Ohm's characteristic to SCLC characteristic. *V*_i_ means that the injected carrier is sufficiently large to overcome the influence of thermal free carriers [33]. Carrier density *n*_0_ can be acquired through the equation *n*_0_ = ε*V*_i_/*er*^*2*^ based on the *V*_i_ [31], where ε is the dielectric permittivity, *e* is the free electron charge, and *r* is the radius of the microrope. Table 1 lists the carrier density *n*_0_ covering the temperature range from 360 down to 100 K. *n*_0_ is 5.09 *×* 10^18^ cm^−3^ for 100 K.

**Table 1:** **Calculated charge carrier density** ***n***_**0**_ **of the CdS microrope at different temperatures**

	**Value**									
*T* (K)	100	120	150	180	200	230	250	300	320	360
*n*_0_ (10^18^ cm^−3^)	5.09	5.64	6.42	6.64	7.12	7.74	8.63	9.96	14.38	16.04

The values of the exponent *M* can be obtained from fitting, as shown in Figure 2b. Here, *M*_1_ represents the value of *M* at lower voltages, indicating Ohm's characteristic of the *I*-*V* curves, and *M*_2_ represents the value of *M* at higher voltages, indicating the SCLC characteristic of the *I*-*V* curves. The transition from *M*_1_ to *M*_2_ means the transition from Ohm's regime to SCLC regime, which depends markedly on the distribution of the trapping levels in energy, because the appearance of SCLC is inhibited until a sufficiently large electric field is applied [34]. *M*_1_ suggests that the concentration of the thermally generated free carriers is superior to the concentration of injected carriers. *M*_1_ is about 1.05 at 360 K, and it increases gradually with decreasing temperature. To our surprise, *M*_1_ decreases sharply from 1.7 to 0.5 ~ 0.6 when the temperature is below 100 K. This obvious deviation from the SCLC theory may be attributed to another conduction mechanism, and it will be discussed in the following context. *M*_2_ implies that the concentration of injected carriers is overwhelmingly large to overcome the influence of thermal free carriers. It is evident that *M*_2_ increases with decreasing temperature possibly due to the enhanced thermal emission of trapped charges into the conduction band [31], revealing the reduction of deeper level traps inside the CdS microrope. For example, *M*_2_ is 2 when the temperature is 200 K, implying the material is in trap-free state according to the SCLC theory. For the temperature lowering from 180 to 100 K, the depth of the trap state increases gradually.

The voltage and temperature dependencies of SCLC are extremely sensitive to the presence of defects, which can be used to characterize the density and energy distribution of the defect states [28]. As we know, trap plays an important role in understanding the *I*-*V* characteristics in solid-state physics; in addition, the corresponding characteristic energy could be extracted from a linear fit to the temperature dependence of *M*_2_. From the explanation by Rose [28], *I ∞ V*^*T*c/*T +* 1^, where *T*_c_ is the characteristic temperature relating to the trap energy distribution; furthermore, the relation *T*_c_/*T +* 1 *= M*_2_ can be obtained from the SCLC theory. The trap energy *E* which is measured from the bottom of the conduction band can be *E = k*_B_*T*_c_, where *k*_B_ is the Boltzmann constant. The *E* is 11.7 meV at 100 K, which increases from 11.7 to 17.5 meV (200 K). For comparison, the trap energy dropped from 0.26 eV before annealing to 0.12 eV after annealing. It was consistent with the explanation that the annealing process reduced the deep level traps inside the individual GaSb nanowire [29]. In addition, Simpkins et al. obtained the trap energy of coaxial AlGaN/GaN nanowires which was 75 meV [31].

**Figure 3:**
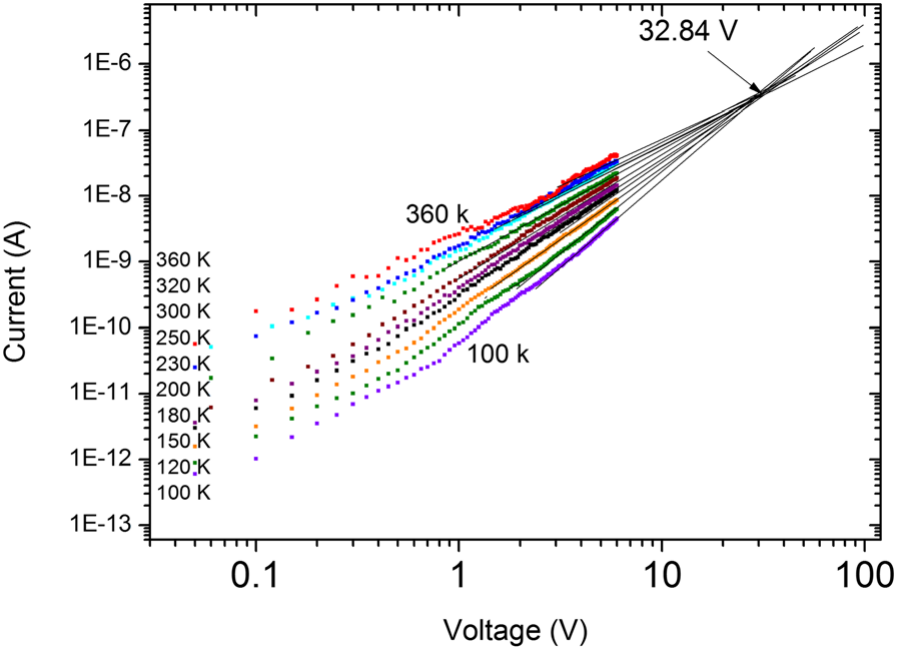
**Crossover voltage** ***v***_**c**_ **at which the** ***I*** **-** ***V*** **curves at various temperatures intersect.** The value of *v*_c_ obtained from fitting is about 32.84 V.

It has been proved that temperature-dependent *I*-*V* characteristic curves should intersect at a crossover voltage, *v*_c_ (Figure 3) [29,31,35], based on this, another corresponding trap characterization, such as trap concentration (trap density) *H* also can be obtained from the relation *v*_c_ *= eHL*^2^/2ε given by Kumar et al. [35], where *L* is the sample length between the two microleads. *v*_c_ is about 32.84 V through extrapolating the *I*-*V* curves to high voltage; thus, the trap concentration is about 4.54 × 10^15^ cm^−3^. For comparison, Simpkins et al. reported that the trap concentration of coaxial AlGaN/GaN nanowires was from 2.5 × 10^16^ to 5.6 × 10^17^ cm^−3^ [31], the value of 106-nm-thick ITO/Alq_3_/Ca devices is 5.9 × 10^18^ cm^−3^ and the value of 550 nm thick is 4.4 × 10^17^ cm^−3^ [35], and the value of GaSb nanowires obtained by Xu et al. was 3.1 × 10^16^ cm^−3^ [29].

The current density is given by the Mott-Gurney Law without any trapping effects [26]. In this theory, the current is assumed to be due to carriers of one sign only, the effect of diffusion is neglected, and the mobility is assumed to be independent of the field [36]. The current density *J* is defined as *J* = *ε*_0_*ε*_r_*μV*^2^/8*d*^3^, where *μ* is the free charge carrier mobility, *d* the distance between the two Pt microleads, *ε*_0_ the vacuum permittivity, and *ε*_r_ the dielectric constant which is about 5 [37]. The carrier mobility is the most important parameter in understanding the transport in some electric devices. For the reason of trap free, only the *I*-*V* characteristic of 200 K is chosen here (the parameter *M*_2_ = 2); thus, the carrier mobility is 87.73 cm^2^ V^−1^ s^−1^, which is in the range of the typical mobility of inorganic semiconductors 10^−5^ ~ 10^3^ cm^2^ V^−1^ s^−1^. For comparison, the mobility of CdTe thin films was 2.539 × 10^−8^ cm^2^ V^−1^ s^−1^ at 303 K [38]. The data of CdSe/ZnS quantum dot composites reported by Hikmet et al. was 1.0 × 10^−6^ cm^2^ V^−1^ s^−1^ at about 423 K [39], and the mobility of Ge_2_Sb_2_Te_5_ layers made by Lebedev et al. was about 10^−3^ cm^2^ V^−1^ s^−1^ at room temperature [40].

### Further discussion: *I*-*V* curves below 80 K

**Figure 4:**
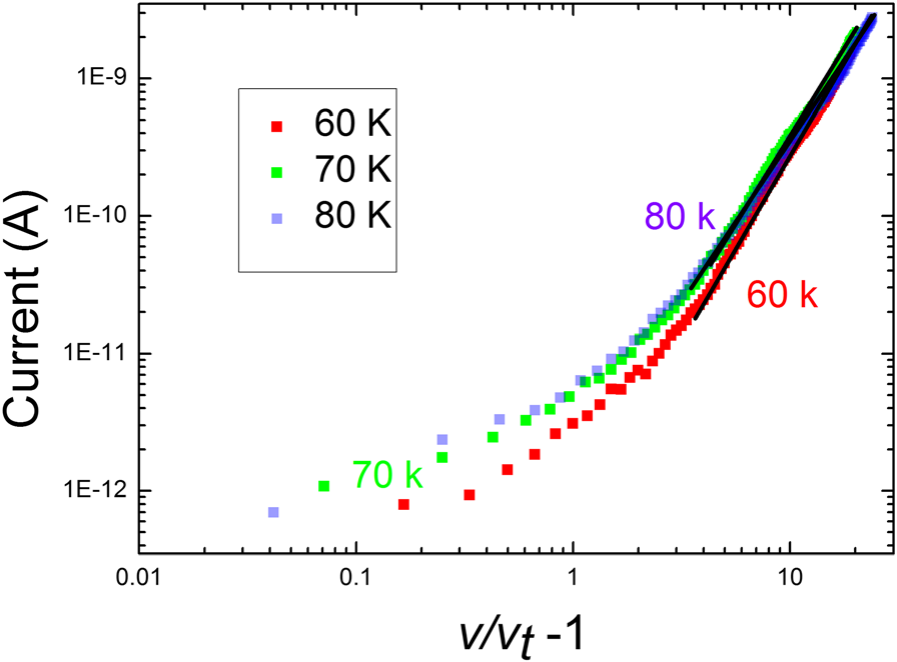
**Current vs (** ***V*** **/** ***V***_**t**_ **− 1) plots at 80, 70, and 60 K.** The straight line is the fitting to equation *I ∞* (*V*/*V*_t_ − 1) ^*ζ*^, *V*_t_ is the threshold voltage. The calculated exponent *ζ* is 2.32 for 80 K, 2.38 for 70 K, and 2.59 for 60 K.

As mentioned above, when the temperature is below 80 K, *M*_1_ decreases sharply from 1.7 to 0.5 ~ 0.6, which deviates from the SCLC theory and may be ascribed to the Coulomb blockade effect [13]. One of the aims of studying Coulomb blockade effects is to investigate the possibility of using them in the construction of new integrated microelectronic devices such as single-electron memories [41], single-electron transistor circuits [42], high-precision Coulomb blockade thermometry [43], supersensitive memories, and data storage [44]. The Coulomb blockade effect was first predicted by Gorter et al. in 1951 [45]. It has been extensively investigated in many fields [46-49]. There is no current below a specific threshold voltage *V*_t_ at lower temperatures; however, charge carriers can tunnel from one dot to another when the charging energy of the device can overcome the thermal energy, leading to a power law dependence while above *V*_t:_*I ∞* (*V*/*V*_t_ − 1) ^*ζ*^ [49]. *ζ* is an exponent depending on the dimensionality of the system, which is 1 for the 1D system and 5/3 or 2 for the 2D system [49]. Clarke et al. obtained *ζ* which was 1.6 for 100-nm-wide multilayer of gold particles [46]. Aleshin et al. acquired *ζ* for R-hel-polyacetylene nanofiber which was 1.78 to 2.14, dependent on temperature only slightly [13]. *ζ* for gold nanowire bundle was 1.36 for the nanowire number about 10, and it was about 2.54 for 200 nanowires [47]. In the present article, it is evident that when the temperature is lowered to less than 80 K, a complete suppression of current below *V*_*t*_, can be observed at a lower voltage; such phenomena should be attributed to the Coulomb blockade effect of charges, for the charges could not overcome charging energies. As the temperature rises, the threshold voltages decrease, similar to other reports [47,50]. In addition, the current increases gradually at higher voltages. This phenomenon is qualitatively consistent with the Coulomb blockade theory. The scatter in Figure 4 is the log-log plots of *I* versus (*V*/*V*_t_ − 1) below 80 K; the line is the fitted curve to equation *I ∞* (*V*/*V*_t_ − 1) ^*ζ*^, from which the *ζ* can be obtained: 2.32 for 80 K, 2.38 for 70 K, and 2.59 for 60 K. *ζ* varies weakly with increasing temperature, which is consistent with the theory that *ζ* is weakly temperature dependent.

**Figure 5:**
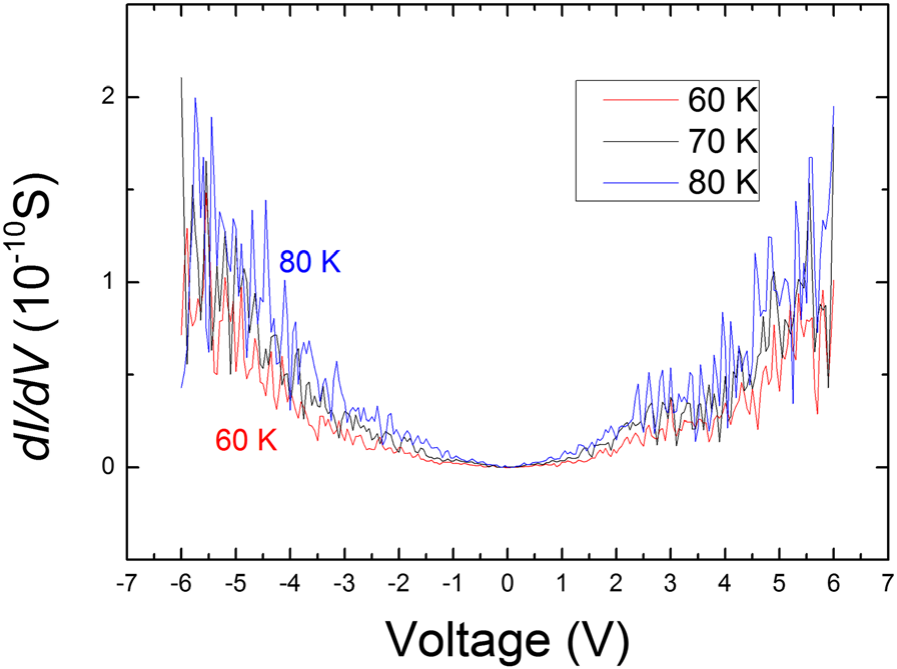
**The differential conductance d** ***I*** **/d** ***V*** **curves at 80, 70, and 60 K.**

The corresponding differential conductance d*I*/d*V* curves could be numerically derived from the corresponding *I*-*V* curves at different temperatures, as shown in Figure 5. Clear oscillations can be observed, due to the periodic modulation of the charging energy. Surprisingly, little oscillation can be seen at a lower voltage, and it seems that the magnitude of oscillations of the three given temperatures is in the same order. However, the magnitude of oscillations increases greatly with increasing voltage. All showed that electron–electron interaction should be taken into account in the SCLC theory especially at lower temperatures.

## Conclusions

In summary, the electrical transport properties of an individual CdS microrope composed of twisted nanowires are studied in the temperature range from 360 down to 60 K. The results show that the *I*-*V* curves can be well fitted by the SCLC theory at higher temperatures. For example, the conduction mechanism is dominated by trap-free space-charge-limited current at 200 K. Trap concentration and carrier mobility are calculated to be 4.54 × 10^15^ cm^−3^ and 87.73 cm^2^ V^−1^ s^−1^ separately. However, the conduction mechanism may be attributed to the Coulomb blockade effect when temperature is below 80 K. It shows that the electron–electron interaction should be taken into account especially at low temperatures in inhomogeneous quasi-1D systems.
